# Vitamin D3 and COVID-19 Outcomes: An Umbrella Review of Systematic Reviews and Meta-Analyses

**DOI:** 10.3390/antiox12020247

**Published:** 2023-01-22

**Authors:** Fausto Petrelli, Simone Oldani, Karen Borgonovo, Mary Cabiddu, Giuseppina Dognini, Mara Ghilardi, Maria Chiara Parati, Daniela Petro’, Lorenzo Dottorini, Carmen Rea, Veronica Lonati, Andrea Luciani, Antonio Ghidini

**Affiliations:** 1 Oncology Unit, ASST Bergamo Ovest, 24047 Treviglio, Italy; 2 Oncology Unit, Casa di Cura Igea, 20129 Milan, Italy

**Keywords:** vitamin D3, COVID-19, infection, meta-analysis, umbrella review

## Abstract

Background: The immune system (innate and adaptive) is influenced by vitamin D3, which affects gene expression and inflammatory pathways. An umbrella review was conducted to evaluate the power and accuracy of data connecting vitamin D3 to the outcomes of COVID-19 infection and to appraise the proof provided by published meta-analyses. Methods: MEDLINE, Embase, and the Cochrane Library were searched from database inception to 31 May 2022. Meta-analyses of prospective or retrospective observational studies and randomized trials were included. Evidence of association was graded according to the established criteria: strong, highly suggestive, suggestive, weak, or not significant. Results: From 74 publications, 27 meta-analyses described five associations between vitamin D3 levels and supplementation and COVID-19 outcomes. Low levels of vitamin D3 were significantly associated with severity (highly suggestive evidence; OR = 1.97 [95% CI, 1.55–2.51], *p* < 0.01; I^2^ = 77%, *p* < 0.01) and mortality risk due to COVID-19 disease (OR = 1.83 [95% CI, 1.55–2.16], *p* < 0.01; I^2^ = 50%, *p* < 0.01). Vitamin D3 supplementation, after a diagnosis of COVID-19 infection, was associated with significantly reduced infection severity (e.g., ICU admission) and mortality. Conclusions: This umbrella review of the available evidence suggests that insufficient vitamin D3 may increase COVID-19 infection risk, severity, and mortality, in addition to showing a highly suggestive association between vitamin D3 supplementation and reduced severity and mortality among infected patients.

## 1. Introduction

Vitamin D modulates the systemic inflammatory response and may actively protect against respiratory tract infections and other diseases [1]. A recent meta-analysis showed that vitamin D supplementation reduces all-cause mortality [2]. In addition, vitamin D supplementation reduces the risk of acute respiratory infections, despite having a small effect size [3]. The outbreak of coronavirus during the last quarter of 2019 resulted in a global pandemic. The virus responsible for the illness was named severe acute respiratory syndrome coronavirus 2 (SARS-CoV-2), and the disease it caused was called coronavirus disease 2019 (COVID-19). COVID-19 infection can range from asymptomatic to mild respiratory tract symptoms to severe pneumonia requiring mechanical ventilation, and may require supportive measures, such as admission to an intensive care unit. The disease has no specific treatment, with options ranging from no intervention to administration of oxygen, steroids, and antivirals.

Multiple systematic reviews and meta-analyses have investigated the correlation between vitamin D3 levels and COVID-19 outcomes. However, the reported associations may not be valid because of inherent biases, such as selective bias, publication bias, or other factors. Additionally, the evidence has not been systematically evaluated; therefore, we conducted a meta-analysis of observational and randomized studies to confirm and grade the burden of data. The purpose of this study was to provide an up-to-date assessment of the evidence, evaluating the strength of the published evidence regarding the association between vitamin D3 levels and supplementation with various COVID-19 outcomes.

## 2. Material and Methods

This study adhered to the Meta-analysis of Observational Studies in Epidemiology (MOOSE) reporting guideline [4].

### 2.1. Literature Search

We searched PubMed, Embase, and the Cochrane Database of Systematic Reviews from the beginning of May 2022. The search terms used were (“meta-analysis”) AND (“vitamin D3” OR “25-hydroxyvitamin D”) AND (“COVID-19”). In addition, we manually screened references to identify eligible articles. A literature search was independently conducted using FP and AG. The differences between the FP and AG were discussed and resolved using AL.

### 2.2. Selection Criteria

Two authors (FP and AG) independently reviewed the full text of potentially eligible articles. Only meta-analyses of epidemiological studies examining the relationship between vitamin D3 and COVID-19 outcomes in adults have been considered. In addition, narrative or systematic reviews without meta-analysis and analyses of the effect of vitamin D3 on other respiratory diseases, conference abstracts, and letters to the editors were excluded. When several meta-analyses from the same authors reported the same health outcomes, we included the meta-analysis with the largest number of studies. In summary, meta-analyses of prospective (observational or randomized) or retrospective studies evaluating the correlation between vitamin levels, according to the cutoff chosen by each author, were analyzed to address three main outcomes: COVID-19 infection risk, infection severity (ICU admissions), and mortality. In addition, meta-analyses with the same criteria as above, exploring the association between vitamin D3 supplementation at infection diagnosis and severe illness (ICU admission or death) in adult patients, were included.

### 2.3. Data Extraction

Data from the included studies were extracted separately by two authors (FP and AG). For each eligible meta-analysis, we extracted the following information: first author, publication year, study design, number of participants and trials, crude or multivariable-adjusted summary risk estimates and corresponding 95% CIs (odds ratios [ORs], risk ratios [RRs], or hazard ratios [HRs]), *p* values of pooled effects, Egger’s test measurement, and I^2^ with *p* for significance. Discrepancies were resolved through discussion. We re-examined the estimated risk of an event for each relationship using random effects models. The three measures of association (OR, RR, and HR) were expected to produce comparable results, as the associated risks of the outcomes we studied were likely to be low. To keep things simple, all risk estimates were explained in terms of odds ratios (ORs).

### 2.4. Assessment of Methodological Quality

Two authors carefully assessed the quality of each meta-analysis using the reliable AMSTAR 2 [5] tool. Based on the scores, four grades were assigned to describe methodological quality: high, moderate, low, and critically low. A high score was assigned when there was no or only one minor defect present, and a moderate score was assigned when there was more than one defect.

### 2.5. Grading of the Evidence

Meta-analyses showing nominally significant associations were categorized into 4 evidence groups: strong, highly suggestive, suggestive, and weak [6]. Associations were considered to be supported by strong evidence if all the following criteria were met: the meta-analysis included >1000 cases, a threshold that provides 80% power for hazard ratios ≥ 1.20 (α = 0.05); the random-effects model had a *p* value ≤10^−6^ (under the assumption that the tests of statistical significance of the effect in each meta-analysis were valid); absence of high heterogeneity (I^2^ < 50%); 95% prediction intervals excluded the null value; and no evidence of small-study effects and excess significance bias. There was strong evidence associated with meta-analyses containing more than one thousand cases, with a random-effects *p*-value of 10^−6^ or lower. The largest study in the meta-analysis was nominally statistically significant. Meta-analyses with more than 1000 cases and a random-effects *p*-value of 10^−3^ or lower yielded suggestive evidence. All other nominally significant associations were considered weak.

### 2.6. Data Analysis

Sensitivity analysis was performed after excluding studies with a significant risk of bias and low-quality levels. When the *p*-value was <0.05, the total effects of the pooled meta-analyses were considered significant. I^2^ and *Q* tests were used to evaluate heterogeneity between studies and Egger’s test was used to calculate publication bias. *p* < 0.1 for both heterogeneity and publication bias was considered significant.

## 3. Results

This umbrella review identified 74 publications and included 27 meta-analyses (36%) describing five associations. Figure 1 in the Supplement lists the 47 articles (65%) that were excluded and the reasons for their exclusion.

Table 1 describes the characteristics of the 27 eligible meta-analyses [7,8,9,10,11,12,13,14,15,16,17,18,19,20,21,22,23,24,25,26,27,28,29,30,31,32,33], which were published between 2021 and 2022.

The median number of studies included in the meta-analysis was 20. The median sample size in the meta-analysis was 11,901. Overall, 12,767,045 patients were included in these 27 meta-analyses.

Using the AMSTAR 2 method (Table 1), we evaluated the methodological quality of five meta-analyses as high (18.5%) and found that nine (33.3%) were of moderate quality. Two meta-analyses (7.4%) were rated as low-quality, and 11 (40.7%) were rated as critically low-quality.

A description and summary of the association between vitamin D3 levels and supplementation with endpoints are presented in Table 2.

There was considerable heterogeneity among the three associations (those that correlated levels with outcomes: I^2^ = 76, 77, and 50%). Neither of the other two associations (vitamin D3 supplementation and outcome) were heterogeneous (I^2^ = 0%). There were small-study effects in 6, 7, 10, 4, and 4 studies for each association (50, 50, 58, 44, and 40%, respectively), so grade levels were downgraded for each association. In the largest study, all associations were statistically significant at *p* < 0.05 for all effect sizes.

Each of the five associations reached statistical significance at *p* < 0.01, suggesting vitamin D3 supplementation or adequate vitamin D3 levels may protect against COVID-19 risk and unfavorable outcomes.

## 4. Grading of Evidence

### 4.1. Susceptibility to Infection

**Figure 2:**
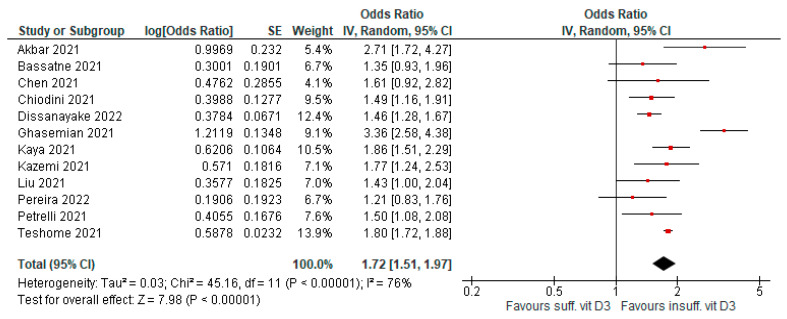
Forest plot showing results for the association of vitamin D3 levels and COVID-19 infection risk.

Low levels of vitamin D3 were associated with a significant risk of COVID-19 infection compared to sufficient levels. Twelve meta-analyses evaluated this outcome (AMSTAR 2 quality level was moderate to high in seven papers). Evidence was graded as highly suggestive (OR = 1.72 [95% CI, 1.51–1.97], *p* < 0.01; I^2^ = 76%, *p* < 0.01; Figure 2).

### 4.2. Risk of Severe Infection (ICU Admission)

**Figure 3:**
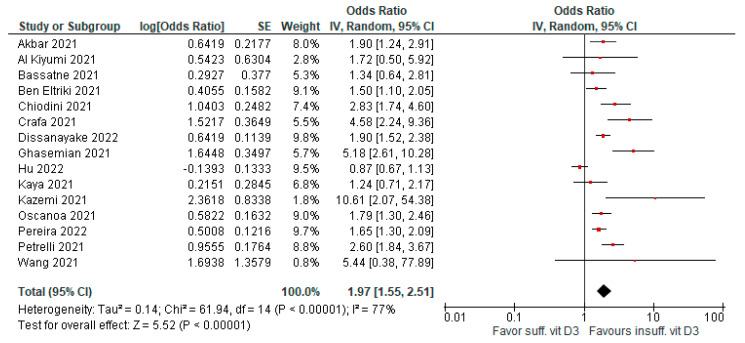
Forest plot showing results for the association of vitamin D3 levels and COVID-19 severity.

Low levels of vitamin D3 were associated with significant risk of severity compared to patients with sufficient levels. Fifteen meta-analyses evaluated this outcome (AMSTAR 2 quality level was moderate to high in seven papers). Evidence was graded as highly suggestive (OR = 1.97 [95% CI, 1.55–2.51], *p* < 0.01; I^2^ = 77%, *p* < 0.01; Figure 3).

### 4.3. Risk of Death

**Figure 4:**
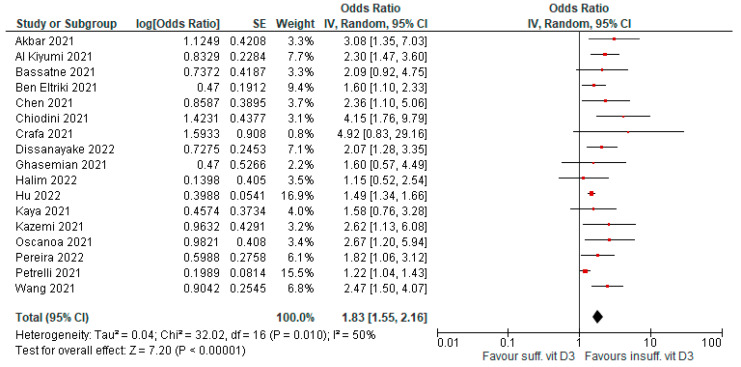
Forest plot showing results for the association of vitamin D3 levels and COVID-19 mortality.

Low levels of vitamin D3 were associated with significant mortality risk compared to patients with sufficient levels. Seventeen meta-analyses evaluated this outcome (AMSTAR 2 quality level was moderate to high in eight papers). Evidence was graded as highly suggestive (OR = 1.83 [95% CI, 1.55–2.16], *p* < 0.01; I^2^ = 50%, *p* = 0.01; Figure 4).

### 4.4. Supplementation to Improve Infection Severity

**Figure 5:**
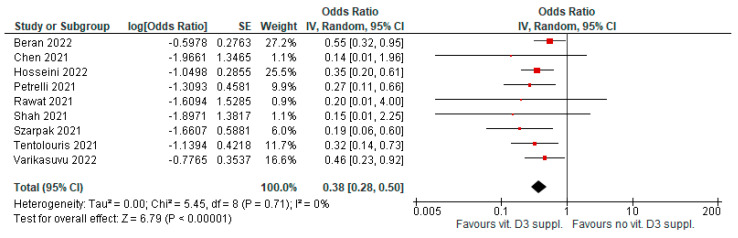
Forest plot showing results for the association of vitamin D3 supplementation and COVID-19 severity.

Vitamin D3 supplementation, after a diagnosis of COVID-19 infection, was associated with significantly reduced infection severity (e.g., ICU admission). Nine meta-analyses evaluated this outcome (AMSTAR2 had a moderate-high level quality in five papers). Evidence was graded as highly suggestive for evidence of publication bias in two meta-analyses (OR = 0.38 [95% CI, 0.28–0.5], *p* < 0.01; I^2^ = 0%, *p* = 0.71; Figure 5).

### 4.5. Supplementation to Improve Infection Mortality

**Figure 6:**
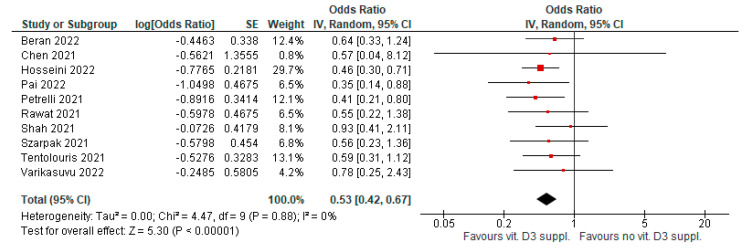
Forest plot showing results for the association of vitamin D3 supplementation and COVID-19 mortality.

Vitamin D3 supplementation, after a diagnosis of COVID-19 infection, was associated with significantly reduced infection mortality. Ten meta-analyses evaluated this outcome (AMSTAR 2 quality level was moderate to high in six papers). Evidence was graded as highly suggestive for evidence of publication bias in three meta-analyses (OR = 0.53 [95% CI, 0.42–0.67], *p* < 0.01; I^2^ = 0%, *p* = 0.88; Figure 6).

### 4.6. Sensitivity Analysis

In studies where the risk of infection and disease severity were correlated with vitamin D3 levels, the strength of association remained unchanged after the exclusion of biased meta-analyses (I^2^ 85 and 51%). After exclusion of low levels, the meta-analysis association became strong for risk of infection and remained highly suggestive of disease severity (I^2^ 41 and 75%). For correlation with mortality, the association became strong after excluding papers that retained significant bias and low-quality levels (I^2^ 0 and 46%). Both outcomes explored in supplementation studies maintained an unchanged grade of association (highly suggestive) after excluding studies with significant bias and low-quality levels (I^2^ 0 and 0%). After considering papers that only calculated adjusted ORs (seven papers), the association between vitamin D3 levels and infection and mortality become strong.

## 5. Discussion

The most common forms of vitamin D supplementation are cholecalciferol (vitamin D3) and ergocalciferol (vitamin D2), which are the precursors of 1,25(OH)_2_D_3_ (the active form of vitamin D). Besides its essential biological functions (classical), such as bone metabolism and calcium and phosphorus homeostasis, vitamin D is also involved in other roles (non-classical) involving immune modulation, lung and muscle function, cardiovascular health, and infectious disease prevention.

Many reports made in Europe, not among those included in these meta-analyses, based on observations made during the first pandemic wave, have suggested an association between vitamin D deficiency, risk of SARS-CoV-2 infection, incidence and severity of COVID-19, and mortality. Speculative observations related to the higher prevalence of hypovitaminosis D among European countries and the very high prevalence of SARS-CoV-2 and COVID-19 infections, especially in the northern regions, have defined the association between the two events without verifying the causal link or excluding causality. Vitamin D status, risk of infection, and development of severe forms of the disease are complex phenomena dependent on innumerable variables whose complex interdependent relationship cannot be described by their mere sum. Therefore, only studies conducted in large cohorts and/or pooled/umbrella analyses can assume epidemiological relevance.

Here, we present the first umbrella review of published meta-analyses on the association between vitamin D3 and COVID-19. We included 27 published meta-analyses, which comprised five summary risk estimates for the association vitamin D3 insufficiency (<30 ng/mL) and supplementation have with COVID-19 outcomes. We found a significant effect size that supported all the statistically significant associations in the primary and sensitivity analyses. There is highly suggestive evidence for an association between low vitamin D3 levels and COVID-19 incidence and disease severity (ICU admission). Furthermore, suggestive evidence exists for positive associations between insufficient vitamin D3 levels and mortality in infected patients (83% increased risk compared with normally replete patients). However, we did not analyze continuous vitamin D3 level/effect associations due to data limitations. Furthermore, after exclusion of biased or low-quality meta-analyses, or those that included unadjusted (crude) ORs, the association with mortality became strong. Finally, we found positive, highly suggestive evidence of an association between vitamin D administration and risk of death reduction.

Vitamin D regulates other cellular functions, in addition to calcium and bone homeostasis. The vitamin D receptor is universally expressed in nucleated cells. A large amount of epidemiological data indicate that the risks of cancer and infectious, autoimmune, and vascular diseases are higher when 25-hydroxyvitamin D (25[OH]D) levels are <20 ng/mL, and that risks decrease with higher 25(OH)D concentrations [34]. However, no convincing randomized trial data has shown that vitamin D supplements can decrease cancer risk or prognosis, decrease the risk or severity of infections or autoimmune diseases, or decrease cardiovascular risks or metabolic diseases. Subjects with normal vitamin D3 levels may be healthier and less prone to many diseases, as well as to COVID-19 infection and potential consequences (e.g., intubation). In particular, in COVID-19 patients, a meta-analysis showed that patients with sufficient vitamin D had significantly lower levels of interleukin-6, C-reactive protein, ferritin, lactate dehydrogenase, fibrinogen, and D-dimer compared to those in the vitamin D deficient group [35].

It is highly plausible that the proposed biological mechanisms explain the observed associations. Several genes involved in the immune system (innate and adaptive immunity) and the downstream inflammatory cascade are influenced by vitamin D, which affects the susceptibility to and severity of infectious diseases. Patients with COVID-19 may benefit from vitamin D supplementation as a preventive and therapeutic agent. Vitamin D binds to its receptor and affects two main pathways: (a) it inhibits pro-inflammatory cytokines, interfering with the TNF-induced NFkB1 pathway, and (b) it activates the Jak-Stat pathway by inducing the expression of interferon-stimulating genes [36,37,38].

More specifically, 1,25(OH)_2_D has antimicrobial activity, as it can induce the expression of cathelicidin and β-defensin 2 proteins with both direct and indirect antimicrobial efficacy (through stimulation of chemotaxis of cells of the immune system, inducing the expression of pro-inflammatory cytokines, resulting in the removal of infected cells in the respiratory tract). The expression of β-defensin [39] is stimulated by vitamin D through the induction of nucleotide-binding oligomerization domain-containing protein 2 (NOD2) 2. Furthermore, 1,25(OH)_2_D inhibits the expression of hepcidin and, therefore, determines the elimination of the hepcidin-mediated block of iron export dependent on ferroportin, resulting in increased efflux of iron from the infected cell and, consequently, the reduction in the availability of this element for microbial growth [40]. Indeed, vitamin D has multiple antimicrobial effects, including stimulation of the barrier function of the intestine [41] and alveolar epithelia [42], production of reactive oxygen species (ROS) [43], neutrophil function [44], and phagocytic and autophagocytic activities (through the induction of the key effectors of autophagy, LC3, beclin 1, and PI3Kγ3) of macrophages [45]. Both the induction of cathelicidin and defensins and stimulation of one of the pro-autophagic pathways in antigen-presenting cells have an important antiviral effect, inhibit virus replication [46], and aid in the clearance of virus particles [47]. In the context of adaptive immunity, calcitriol limits the activation of T lymphocytes [48], induces the expression of regulatory phenotypes (Tregs) that mediate immune tolerance, and limits abnormal immune responses and phenotypic shifts from T helper Th1/Th17 to Th2 (from pro-inflammatory to regulatory) [49]. The effectiveness of vitamin D is a function of the activity of its receptor, VDR. Single nucleotide polymorphisms (SNPs) in the VDR gene affect protein responsiveness and have been associated with numerous immune dysfunctions. Compared to the CT and CC genotypes, the TT genotype of the FokI polymorphism has been associated with a greater risk of respiratory syncytial virus (SCV) infections [50].

The attack/replication sequence into human cells explains another possible therapeutic role of vitamin D. Through its spike protein, SARS-CoV-2 enters the human body via its cognate angiotensin, converting enzyme-2 (ACE-2) receptor on the cells of the lungs and other organs [51]. As soon as the viral RNA enters the host cell, it replicates continuously, using the host’s polymerase system. This leads to severe pathological changes and, finally, cell death. The virus uses multiple mechanisms to evade the host immune system, including excessive stimulation of the renin-angiotensin system (RAS) [52]. SARS-CoV-2 interferes with and modulates the RAS system, resulting in an excess of angiotensin II being generated, activating the cytokine storm, and downregulating the host’s immune system. Dysfunctional immune responses in some COVID-19 cases lead to overproduction of pro-inflammatory cytokines in circulation that trigger acute respiratory distress syndrome (ARDS) and eventually death. Vitamin D has been hypothesized to play a role in acute respiratory distress syndrome (ARDS). ACE2, which, as previously reported, serves as the docking site for viral protein S, converts angiotensin II (Ang-II) to angiotensin 1–7 [Ang(1–7)]. The latter has vasodilator, anti-inflammatory, and protective effects against organ damage actions [53]. Following binding to protein S, the ACE2-viral particle complex is internalized and, therefore, the enzymatic activity of ACE2 is downregulated. Downregulation of ACE2 is associated with an abnormal inflammatory response that can cause tissue damage, leading to further downregulation of ACE2. This process can result in acute respiratory distress syndrome (ARDS) [54,55]. Vitamin D has a protective role against ARDS, given the ability to inhibit the expression of renin and the activity of the ACE/Ang-II/AT1R axis and to stimulate, instead, the ACE2/Ang-(1–7)/MasG (G protein-associated Mas receptor). Thus, vitamin D acts as a negative endocrine modulator of the renin-angiotensin-aldosterone system (RAAS) [56,57,58].

An abnormal inflammatory response resulting from SARS-CoV-2 infection is responsible for the development of COVID-19 and, in some cases, for manifestations of increasing severity. The so-called “cytokine storm”, characterized by the massive and sustained release of pro-inflammatory cytokines (IL-1, IL-6, TNFα, and IFNγ), is responsible for the symptoms and organ damage (particularly affecting the lungs and heart). In severe COVID-19 cases, IL-6 levels were 2.9 times higher than in cases with less severe disease. This evidence supports the role of vitamin D in reducing cytokine storms by inducing anti-inflammatory mediators (IL-10, IL-4, and TGFβ). Furthermore, 1,25(OH)_2_D can reduce the hyperinflammatory response and, therefore, the manifestations of COVID-19 by enhancing the expression of anti-inflammatory and regulatory phenotypes Th2 and T-reg at the expense of pro-inflammatory Th1/Th17, which is also more prominently involved in the cytokine storm [59].

The results of this study suggest a positive association between vitamin D3 supplementation and the severity and mortality of COVID-19 (a reduction in events by approximately 60% and 50%, respectively), as described by Albergamo et al. [60]. However, its dosage and timing are yet to be determined. A recent randomized trial found that the administration of vitamin D did not affect the severity of COVID-19 [61]. In this trial, a single dose of 200 000 IU vitamin D was administered to hospitalized patients with moderate or severe disease. Whether high-dose bolus or continuous low-dose administration is the preferred regimen remains unclear. In addition, whether patients with only true deficiency (<20 ng/mL) or the whole COVID-19 population should be treated with cholecalciferol is still unknown. Current recommendations suggest treating patients with vitamin D3 insufficiency with daily doses of 400–800 units for up to 3 months [62]. Only those with severe vitamin D3 deficiency (<10 ng/mL) may be offered higher doses; however, in general, higher monthly doses can increase the risk of falls in older adults [63]. NICE has proposed specific guidelines related to vitamin D3 administration during the COVID-19 pandemic. In this document, all adults (including women who are pregnant or breastfeeding), young people, and children older than 4 years should consider taking a daily supplement containing 400 units of vitamin D between October and early March because people do not produce enough vitamin D from sunlight in these months [64].

Other micronutrients or vitamin D agents may also play a role in this disease. In particular, compounds such as vitamin C and zinc may play a role; however, Beran et al. [27] found that vitamin C and zinc might not reduce the mortality or severity of COVID-19.

This study has several limitations. First, we must acknowledge that most of the studies examined were retrospective in nature, and a few made adjustments for common variables (e.g., age and pre-existing chronic diseases). In addition, few randomized controlled trials that are capable of managing confounding factors due to their design are available for supplementation. Additionally, many of the included studies showed publication bias based on Egger’s test and a moderate to low quality according to the AMSTAR 2 checklist, leading to the recommendation that researchers follow methodology guidelines to ensure the methodological quality of future meta-analyses. This resulted in the evidence for two associations being downgraded from strong to highly suggestive. When studies with intrinsic bias or low quality were excluded, vitamin D3 levels strongly correlated with COVID-19 mortality. Furthermore, we were unable to perform subgroup analyses due to the absence of data (e.g., sex, age, previous use of vitamin D3, comorbidities, and country of origin).

The following considerations can be made based on the results of these studies. Even if further controlled studies are needed, vitamin D appears to be more effective against COVID-19 (both due to the speed of negativization and the benign evolution of the disease in case of infection) if administered prior to the manifested infection, especially in elderly, frail, and institutionalized subjects. The minimum optimal plasma target of 25(OH)D to be reached in the preventive setting would be ≥30 ng/mL, for which it is necessary to administer high doses of cholecalciferol, also in relation to the basal levels of the patient, up to 4000 IU/day [37,65,66]. With the preventive administration of oral cholecalciferol (up to 4000 IU/day), the use of vitamin D, which, even at high doses, does not present substantial side effects, is useful for correcting a situation of specific general deficiency in the population, especially in winter, regardless of SARS-CoV-2 infection [67]. Additionally, higher parenteral doses of vitamin D3 could have an effect on the final outcome, as bypassing liver vitamin D3 will also be available to CYP11A1 for metabolism in organs expressing this enzyme [68]. In addition to targeting COVID-19 through both nuclear receptor-dependent and-independent mechanisms, new CYP11A1-derived active forms of vitamin D and lumisterol can be used as antiviral drugs and supplements to prevent and attenuate it [69].

## 6. Conclusions

In conclusion, the results of the present study support and reaffirm the recommendation for vitamin D3 supplementation as helpful in preventing COVID-19 (particularly for elderly or frail people, housebound individuals, or individuals living in nursing homes and who spend a great deal of their time indoors due to COVID-19), while emphasizing its potential anti-inflammatory and immunomodulatory properties to lessen the severity and mortality of infected individuals. Additionally, this evidence supports the possibility of monitoring vitamin D3 levels, especially in high-risk populations. However, additional research is necessary to determine the optimal dose, timing, and duration of vitamin D3 therapy for infected patients.

## Figures and Tables

**Figure 1:**
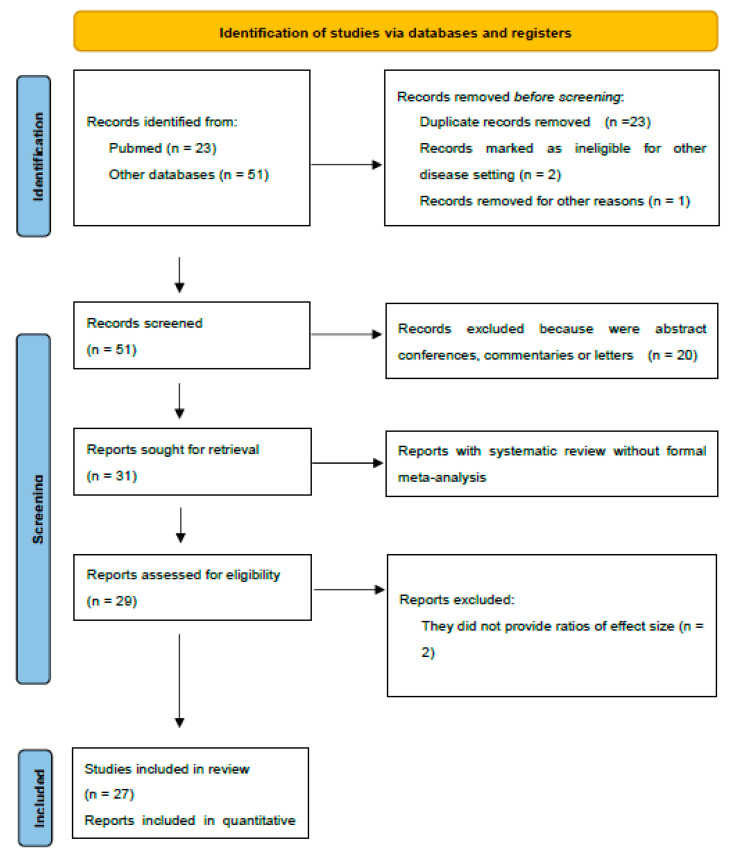
Flow diagram of the included studies.

**Table 1:** Characteristics of the included studies.

Author/ Year	N° Studies/ N° pts	Type of Studies	Time of Search	Vitamina D3 Cutoff (ng/mL)	Vitamin D3 Dose/Timing	Outcome Evaluated	Severity Definition	Type of Metric/ Type of Analysis	Small Study Effect (*p* Egger Test)	Overall Quality Assessment (AMSTAR2)
Akbar/2021	14/999,179	9 Retrosp observational	Up to 9 December 2020	20–30	-	- Risk of infection and VitD levels	Criteria for severe CAP (ICU, MV)	OR/-	Severity (*p* 0.047) Mortality (*p* 0.046)	Moderate
		2 Prosp Observational				- Risk of severity and VitD levels				
		2 Cross-Sect				- Risk of death and VitD levels				
		1 Observational study								
Al Kiyumi/2021	43/254,963	18 Retrosp	Until 20 December 2020	20	-	- Prevalence of VDD/VDI in patients with COVID-19	ARDS, severe radiology, ICU admission, O_2_ therapy, ventilator support	OR/MVA	Severity (*p* 0.071) Mortality (*p* 0.023)	Moderate
		11 Cross-Sect				- Severity of COVID-19				
		8 Prosp				- Case-fatality rate				
		3 Case-control								
		1 Observational study								
		1 pilot study								
		1 Case series								
Bassatne/2021	31/8209	15 Cross-Sect	Until 20 January 2021	20	357 to 60,000 IU die/from one week to 12 months	Association of low serum 25(OH)D with COVID-19 related health outcomes:	-	RR/-	Not performed	High
		11 Cohort				- Mortality				
		4 Case-control				- ICU admission				
						- Invasive ventilation				
						- Non-invasive ventilation				
						- Hospitalization				
						- Length of hospital stay				
						- Disease severity				
						- ARDS				
						Serum 25(OH)D levels in COVID-19 infected patients compared to those not infected				
Ben-Eltriki/ 2021	24/3637	7 Retrosp observational	Up to 30 March 2021	30	-	- All-cause mortality	Total number of severe cases, hospital duration, MV	RR/-	Not reported	Critically low
		6 Prosp cohort				- COVID-19 severity				
		3 Cross-Sect				- Difference in biological markers and disease severity				
		2 Retrosp								
		2 Prosp observational								
		2 Cohort								
		2 Case-control								
Beran/2022	14/3497	4 Retrosp cohort	Through 5 December 2021	-	Various	-Risk of mortality	-	RR/-	(*p* 0.047)	High
		4 RCT				- Intubation rate				
		3 Non-RCT								
		2 Cross-Sect								
		1 Case-control								
Chen/2021	13/536,338	10 Retrosp cohort	Up to 5 June 2021	20–30	Various	- Effect of low VitD level on COVID-19	ICU admission	OR/MVA	Not performed	Moderate
		2 RCT				- Effect of VitD supplements on ICU admission or death				
		1 Prosp C								
Chiodini/ 2021	54/ 1,403,715	37 Retrosp	Until 31 March 2021	25	-	- VitD status as a predictor of in-hospital COVID-19 severity	ICU admission	OR/-	Severity (*p* 0.816) Mortality (*p* 0.110)	Moderate
		17 Prosp				- VitD and SARS-CoV-2 infection or COVID-19 related hospitalization				
Crafa/2021	29/380,172	11 Retrosp	Up to January 2021	20	-	- 25(OH)D levels in patients SARS-CoV-2 pos or neg	Severe pneumonia, ICU, severe radiology or combo	OR/-	Not reported	Moderate
		6 Prosp				- 25(OH)D levels in patients with severe or non-severe COVID-19				
		5 Case-control				- 25(OH)D levels in COVID-19 patients who died compared to those discharged				
		3 Observational study				- Risk of severe COVID-19 in patients with VitD deficiency				
		1 pilot study				- Mortality risk in patients with VitD deficiency				
		1 Cohort								
		1 Cross-Sect								
		1 Popul-based								
Dissanayake/ 2022	72/ 1,975,551	28 Retrosp cohort	-	Various	-	- Susceptibility to infection	Hospitalization, hypoxia (O_2_, non/invasive ventilation, ARDS or combo), death or composite	OR/-	(*p* 0.0001)	Moderate
		26 Case-control				- Risk of developing severe COVID-19				
		17 Prosp cohort				- Mortality				
						- VitD in the treatment of COVID-19				
Ghasemian/ 2021	23/11,901	9 Retrosp cohort	Up to 18 December 2020	20–30	-	- Frequency of VitD status in	-	OR/-	Severity (*p* 0.14) Mortality (*p* 0.62)	Critically low
		5 Case-control				COVID-19 patients				
		4 Cross-Sect				- Mean serum 25(OH)D concentration				
		4 Prosp cohort				- VitD deficiency and SARS-CoV-2 infection				
		1 RCT				- VitD deficiency and COVID-19 severity				
						- VitD deficiency and				
						COVID-19 mortality				
Halim/2022	11/1424	4 Retrosp cohort	Up to the year 2021	20	-	- VitD and COVID-19 severity	Various	OR/-	Not reported	Critically low
		4 Cohort				- VitD and COVID-19 mortality				
		3 Cross-Sect								
Hosseini/2022	23/ 5,870,189	9 RCT	Up until January 2022	25	Various	- Risk of COVID-19 infection (primary prevention	-	RR/-	Risk of bias detected in some studies	High
		6 Retrosp cohort				studies on uninfected individuals)				
		4 Prosp cohort				- Hospital admission (secondary prevention studies on mild COVID-19 cases)				
		3 Retrosp				- ICU admission and mortality rate (tertiary prevention studies on hospitalized				
		1 Case-control				COVID-19 patients)				
Hu/2022	20/12,806	6 Retrosp	On 1 May 2021	25	-	- Effect of VitD serum concentration on mortality	ICU admission, ventilator support, length of hospital stay	RR/-	High risk	Low
		4 Retrosp observational				- Effect of VitD serum concentration on ICU admission				
		4 Prosp observational				- Effect of VitD serum concentration on ventilator support requirement				
		2 Retrosp cohort				- Length of hospital stay				
		2 Cross-Sect								
		1 Prosp								
		1 Prosp cohort								
Kaya/2021	21/205,869	11 Case-control	Between 1 January and 15 December 2020	20	-	- VitD and COVID-19 infection	-	OR/-	Severity (*p* 0.064) Mortality (*p* 0.911)	Low
		9 Cohort				- VitD and COVID-19 severity				
		6 Cross-Sect				- VitD and COVID-19 mortality				
Kazemi/2021	39/13,333	11 Case-control	Up to 26 November 2020	Various	-	- Association of 25(OH)D status with SARS-CoV-2 infection	Various	OR/-	(*p* 0.002)	High
		9 Cross-Sect				- Association of VitD status with COVID-19 severity				
		6 Retrosp cohort				- Composite severity				
		4 Retrosp observational				- ICU admission or stay				
		3 Retrosp				- Pulmonary complications				
		3 Prosp				- Hospitalization				
		3 Descriptive				- Concentration of 25(OH)D between severe and less				
		3 Cohort				- severe status of disease				
		2 Prosp cohort				- Inflammatory markers				
		2 Quasi-exp				- Mortality				
		2 RCT								
Liu/2021	10/361,934	10 Case-control	To 25 September 2020	Various	-	- Association between	-	OR/-	(*p* 0.001; *p* 0.009)	Moderate
						VitD deficiency or insufficiency and COVID-19 infection				
						- VitD levels in COVID-19-positive and-negative participants				
Oscanoa/2021	23/2692	11 Case-control	Between December 2019 and December 2020	Various	-	- Mortality and severity	ICU admission, ARDS and/or need for MV	RR/-	Not reported	Critically low
		5 Cross-Sect				- proportions in COVID-19 patients with 25(OH)D deficiency				
		5 Cohort								
		2 Observational study								
Pai/2022	13/2933	10 Observational study	Until 8 June 2021	Various	Various	- ICU admission and/or mortality in COVID-19 patients receiving VitD supplementation	-	OR/-	Risk of bias detected in some studies	High
		3 RCT								
Pereira/2022	25/8176	9 Cohort	Up to 9 October 2020	50	-	- Prevalence of VitD deficiency in severe cases of COVID-19	-	OR/-	Risk of bias detected in some studies	Critically low
		7 Retrosp								
		6 Cross-Sect								
		1 Prosp cohort								
		1 Popul-based								
		1 Case series								
Petrelli/2021	43/612,601	24 Retrosp	Until 31 January 2021	15–30	Various	- Association between VitD and risk, severity, and mortality for COVID-19 infection	Various	OR/MVA	(*p* 0.04)	Critically low
		7 Prosp								
		4 Case-control								
		2 Prosp cohort								
		2 Cross-Sect								
		1 Observational								
		1 Registry data								
		1 Popul-based								
		1 RCT								
Rawat/2021	5/467	3 RCT	Until 18 May 2021	-	Various	- Mortality	ICU admission, MV	RR/-	Risk of bias detected in some studies	Critically low
		2 Quasi-Exp				- Mechanical ventilation				
						- Admission to ICU				
						- Acute markers				
Shah/2021	3/532	2 RCT	Between December 2019 and 17 December 2020	-	Various	- ICU admission	ICU admission, need for MV, mortality	OR/MVA	Severity (*p* 0.253) Mortality (*p* 0.138)	Critically low
		1 Retrosp case-control				- Death				
						- Hospital length of stay				
						- Mechanical ventilation requirement				
						- Serum level of VitD and				
						- biomarkers				
Szarpak/2022	8/2322	3 Observational	Until 10 July 2021	-	Various	- Primary end points were 14-day and in-hospital mortality.	-	OR/-	Risk of bias detected in some studies	Critically low
		1 Retrosp cohort				- Secondary end points were ICU admission, need of mechanical ventilation,				
		1 Retrosp case-control				- radiological improvement and secondary infection incidence				
		1 Quasi-exp								
		1 Pilot study random								
		1 RCT								
Tentolouris/ 2021	9/278	2 Cohort	On 26 March 2021	-	Various	- Mortality	Various	OR/-	Severity (*p* 0.011) Mortality (*p* 0.676)	Moderate
		1 RCT				- ICU admissions				
		1 pilot study random								
		1 Quasi-exp								
		1 Prosp observ. study								
		1 Prosp cross-Sect								
		1 Case-control								
		1 Retrosp cross-Sect								
		1 Retrosp								
Teshome/ 2021	14/91,120	5 Cohort	From 15 May 2020 to 20 December 2020	-	-	- Risk of developing COVID-19 infection among VDD and normal VitD levels	-	OR/-	(*p* 0.764) *	Critically low
		5 Case-control								
		3 Cross-Sect								
		1 Interim audit								
Varikasuvu/ 2022	6/551	6 RCT	To 5 August 2021	-	Various	- Severity	-	RR/-	(*p* 0.14)	Moderate
						- ICU care				
						- Mortality				
						- Seropositivity				
						- RT-PCR positivity				
Wang/2021	17/2756	17 Observational	From 1 January 2019 to 3 December 2020	12–25	-	- Mortality	Mortality, hospital admission, lenght of hospital stay	OR/-	High risk	Critically low
						- Hospital admission				
						- Length of hospital stay				
						- ICU admission				

ICU, intensive care unit; CAP, community-acquired pneumonia; ARDS, acute respiratory distress syndrome; MV, mechanical ventilation; VDD/VDI, vitamin D deficiency/insufficiency; OR, odds ratio; RR, risk ratio; MVA, multivariate analysis; RCT, randomized controlled trial; Case-control, case-control study; Cross-Sect, cross-sectional study; Quasi-Exp, quasi-experimental study; Popul-based, population-based study; Retrosp, retrospective; Prosp, prospective; *, Begg’s test.

**Table 2:** The strength of epidemiologic evidence of the 5 unique health outcomes.

Endpoint	Author/Year	N° Studies	Effect Size (95% CI)	Heterogenity (I^2^, *p*)	Overall Effect
COVID-19 infection	Akbar/2021	6	OR = 2.71 [1.72, 4.29]	92.6%, 0.001	OR = 1.72 (95% CI 1.51–1.97) *p* < 0.01; I^2^ = 76%, *p* for heterogeneity < 0.01 Highly suggestive association
	Bassatne/2021	3	RR = 1.35 (0.93–1.96)	76%, 0.11	
	Chen/2021	3	RR = 1.61 (0.92–1.8)	92%, 0.09	
	Chiodini/2021	7	OR = 1.49 (1.16–1.91)	79%, 0.1	
	Dissanayake/2022	19	OR = 1.46 (1.28–1.65)	92%, <0.001	
	Gasemian/2021	3	OR = 3.36 (2.58–4.37)	NR	
	Kaya/2021	8	OR = 1.64 (1.32–2.04)	85%, <0.01	
	Kazemi/2021	3	OR = 1.77 (1.24–2.53)	44%, 0.16	
	Liu/2021	4	OR = 1.43 (1–2.05)	64%, 0.03	
	Pereira/2022	4	OR = 1.21 (0.83–1.6)	78%, 0.003	
	Petrelli/2021	7	OR = 1.5 (1.08–2.08)	95%, <0.001	
	Teshome/2021	8	OR = 1.8 (1.72–1.88)	71%, <0.001	
Severe COVID-19 infection	Akbar/2021	5	OR = 1.90 [1.24, 2.93]	64.2%, 0.02	OR = 1.97 (95% CI 1.55–2.51) *p* < 0.01; I^2^ = 77%, *p* for heterogeneity < 0.01 Highly suggestive association
	Al Kiyumi/2021	5	OR = 3.38 (1.94–5.87)	67%, <0.01	
	Bassatne/2021	7	RR = 1.34 (0.64–2.79)	0, 0.43	
	Ben Eltriki/2021	12	RR = 1.5 (1.1–2.05)	92%, 0.01	
	Chiodini/2021	10	OR = 2.83 (1.74–4.6)	84%, 0.94	
	Crafa/2021	8	OR = 4.58 (2.24–9.36)	81%, 0.001	
	Dissanayake/2022	36	OR = 1.9 (1.52–2.38)	81%, <0.001	
	Gasemian/2021	13	OR = 5.18 (2.61–10.31)	NR	
	Hu/2022	3	RR = 0.87 (0.67–1.14)	0, 0.31	
	Kaya/2021	9	OR = 1.24 (0.71–2.17)	92%, <0.01	
	Kazemi/2021	6	OR = 10.61 (2.07–54.38)	90, <0.01	
	Oscanoa/2021	4	RR = 1.79 (1.3–2.46)	81%, <0.001	
	Pereira/2022	10	OR = 1.65 (1.3–2.09)	35%, 0.12	
	Petrelli/2021	17	OR = 2.6 (1.84–3.67)	87%, <0.001	
	Wang/2021	4	OR = 5.44 (0.38–78.42)	83%, NR	
COVID-19 mortality	Akbar/2021	8	OR = 3.08 [1.35, 7.00]	80.3%, 0.001	OR = 1.83 (95% CI 1.55–2.16) *p* < 0.01; I^2^ = 50%, *p* for heterogeneity = 0.01 Highly suggestive association
	Al Kiyumi/2021	4	OR = 2.3 [1.47–3.6]	0, 0.07	
	Bassatne/2021	7	RR = 2.09 (0.92–4.77)	76%, 0.08	
	Ben Eltriki/2021	18	RR = 1.6 (1.1–2.32)	68%, 0.01	
	Chen/2021	4	RR = 2.36 (1.1–5.06)	0%, 0.71	
	Chiodini/2021	19	OR = 4.15 (1.76–9.79)	44%, 0.33	
	Crafa/2021	9	OR = 4.92 (0.83–29.31)	94%, <0.001	
	Dissanayake/2022	20	OR = 2.07 (1.28–3.35)	73%, 0.003	
	Gasemian/2021	7	OR = 1.6 (0.57–4.45)	NR	
	Halim/2022	5	OR = 1.15 (0.52–2.49)	66%, 0.34	
	Hu/2022	20	RR = 1.49 (1.34–1.66)	83%, 0.88	
	Kaya/2021	5	OR = 1.58 (0.76–3.28)	83%, <0.01	
	Kazemi/2021	8	OR = 2.62 (1.13–6.08)	83%, <0.001	
	Oscanoa/2021	4	RR = 2.67 (1.2–5.94)	83%, <0.001	
	Pereira/2022	5	OR = 1.82 (1.06–2.58)	59%, 0.04	
	Petrelli/2021	19	OR = 1.22 (1.04–1.43)	74%, 0.01	
	Wang/2021	12	OR = 2.47 (1.5–4.05)	30%, NR	
Effect of supplementation of vitamin D3 on COVID-19 infection severity	Beran/2022	6	RR = 0.55 (0.32–0.97)	48%, 0.04	OR = 0.38 (95% CI 0.28–0.5) *p* < 0.01; I^2^ = 0%, *p* for heterogeneity = 0.71 Highly suggestive association
	Chen/2021	2	OR = 0.14 (0–4.9)	90%, 0.28	
	Hosseini/2022	7	RR = 0.35 (0.20–0.62)	75%, <0.001	
	Petrelli/2021	6	OR = 0.27 (0.11–0.66)	49%, 0.004	
	Rawat/2021	2	RR= 0.2 (0.01–4.26)	89%, 0.3	
	Shah/2021	3	OR = 0.15 (0.01–1.45)	82%, NR	
	Szarpak/2021	5	OR = 0.19 (0.06–0.54)	77%, 0.002	
	Tentolouris/2021	6	OR = 0.32 (0.14–0.71)	60%, 0.028	
	Varikasuvu/2022	6	RR = 0.46 (0.23–0.93)	52%, 0.06	
Effect of supplementation of vitamin D3 on COVID-19 infection mortality	Beran/2022	9	RR = 0.64 (0.33–1.27)	77%, 0.25	OR = 0.53 (95% CI 0.42–0.67) *p* < 0.01: I^2^ = 0%, *p* for heterogeneity = 0.88 Highly suggestive association
	Chen/2021	2	OR = 0.57 (0.04–7.78)	64%, 0.67	
	Hosseini/2022	11	RR = 0.46 (0.30–0.7)	54%, <0.001	
	Pai/2022	7 *	OR = 0.35 (0.14–0.85)	67%, 0.03	
	Petrelli/2021	7	OR = 0.41 (0.21–0.81)	72%, 0.01	
	Rawat/2021	4	RR= 0.55 (0.22–1.39)	58%, 0.21	
	Shah/2021	3	OR = 0.93 (0.41–2.11)	21%, 0.27	
	Szarpak/2021	7	OR = 0.56 (0.23–1.37)	74%, 0.2	
	Tentolouris/2021	9	OR = 0.59 (0.31–1.12)	62%, 0.006	
	Varikasuvu/2022	4	RR = 0.78 (0.25–2.4)	48%, 0.03	

*, 2 studies reported ICU admission and ICU admission or mortality as outcomes.
